# Chemical tools for profiling the intracellular ADP-ribosylated proteome†

**DOI:** 10.1039/d4cb00043a

**Published:** 2024-05-22

**Authors:** Simeon D. Draganov, Michael J. Gruet, Daniel Conole, Cristina Balcells, Alexandros P. Siskos, Hector C. Keun, Dorian O. Haskard, Edward W. Tate

**Affiliations:** a Department of Chemistry, Molecular Sciences Research Hub, Imperial College London London W12 0BZ UK e.tate@imperial.ac.uk; b Department of Surgery and Cancer, Institute of Reproductive and Developmental Biology, Imperial College London London W12 0HS UK; c Faculty of Medicine, National Heart and Lung Institute, Imperial Centre for Translational and Experimental Medicine, Imperial College London London W12 0HS UK

## Abstract

The post-translational modification (PTM) ADP-ribosylation plays an important role in cell signalling and regulating protein function and has been implicated in the development of multiple diseases, including breast and ovarian cancers. Studying the underlying mechanisms through which this PTM contributes towards disease development, however, has been hampered by the lack of appropriate tools for reliable identification of physiologically relevant ADP-ribosylated proteins in a live-cell environment. Herein, we explore the application of an alkyne-tagged proprobe, 6Yn-ProTide-Ad (6Yn-Pro) as a chemical tool for the identification of intracellular ADP-ribosylated proteins through metabolic labelling. We applied targeted metabolomics and chemical proteomics in HEK293T cells treated with 6Yn-Pro to demonstrate intracellular metabolic conversion of the probe into ADP-ribosylation cofactor 6Yn-NAD^+^, and subsequent labelling and enrichment of PARP1 and multiple known ADP-ribosylated proteins in cells under hydrogen peroxide-induced stress. We anticipate that the approach and methodology described here will be useful for future identification of novel intracellular ADP-ribosylated proteins.

## Abstract 2

6Yn-Pro is a cell-permeable proprobe successfully undergoing metabolic conversion into 6Yn-NAD^+^ for subsequent intracellular labelling of ADP-ribosylated proteins.

## Graphical abstract

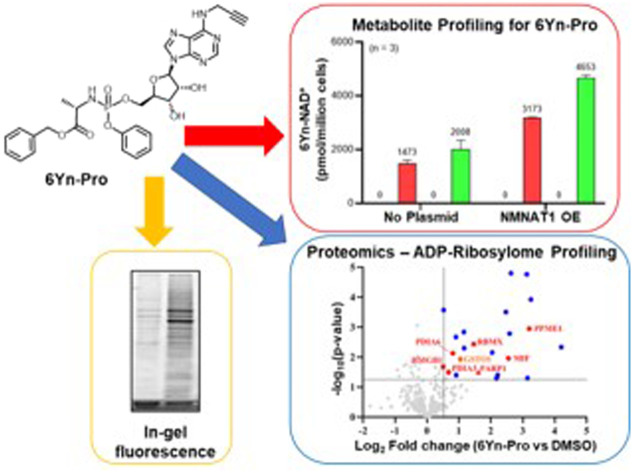

## Introduction

ADP-ribosylation is a type of post-translational modification most commonly found on proteins.^1,2^ It is catalysed by poly(ADP-ribose) polymerases (PARPs), a family of 17 enzymes, which catalyse covalent coupling of ADP-ribose units, derived from the cellular cofactor nicotinamide adenine dinucleotide (NAD^+^), to protein substrates.^3,4^ PARPs are broadly divided into two classes: mono-ADP-ribosylating (MARylating) PARPs, which catalyse the addition of a single ADP-ribose unit to a protein target, a process known as mono-ADP-ribosylation (MARylation), and poly-ADP-ribosylating (PARylating) PARPs, which catalyse the addition of multiple (up to 200) ADP-ribose units to a protein target, a process known as poly-ADP-ribosylation (PARylation).^4,5^ PARPs have been implicated in a very wide range of biological processes including repair of single- and double-strand DNA breaks as part of the DNA damage response (DDR),^6–8^ RNA metabolism by altering localisation and activity of RNA-binding proteins,^9^ antiviral response by repressing viral replication through degradation of viral RNA/proteins and amplification of the interferon response,^10–14^ and neuronal development by regulating dendrite morphogenesis.^15^ In addition to their roles in regulating essential biological processes PARPs have been implicated in development of disease states such as breast and ovarian cancer, through upregulation of ADP-ribosylation activity.^16–20^ Elucidating the underlying mechanisms linking PARP catalytic activity and disease development is of vital importance for development of targeted treatments, for which identification and study of the protein substrates of ADP-ribosylation in relevant disease models represents an important step.

In recent years, several approaches have been developed to study the biological consequences of ADP-ribosylation through identification of the ADP-ribosylated proteome, and for dissecting the biological roles of individual PARPs. NAD^+^ analogues incorporating a click chemistry tag allow for functionalisation of labelled proteins *via* click chemistry conjugation to a fluorophore and/or biotin.^21–35^ This breakthrough enables the use of more complex analytical techniques to study ADP-ribosylation, including visualisation of the distribution of ADP-ribosylation events in fixed cells *via* fluorescence microscopy.^24–27^ Furthermore, biotinylation of ADP-ribosylated proteins allows for their selective enrichment from the cellular proteome, and coupled with liquid chromatography-tandem mass spectrometry (LC-MS/MS), has led to the identification of hundreds of putative ADP-ribosylation targets.^23,30–35^

A major limitation on the use of NAD^+^ analogues in the study of ADP-ribosylation dynamics and identification of relevant protein substrates is their inability to passively cross cell membranes. Studies employing NAD^+^ analogues have therefore been largely confined to experiments involving cell lysates or involve the use of carrier peptides,^24^ transfection reagents^26^ or transiently permeabilised cells^27^ to deliver NAD^+^ analogues intracellularly, each of which is limited by disruption of the cell membrane. Two previous studies employing clickable adenosine precursors for the identification of intracellular ADP-ribosylation targets *via* metabolic conversion into the corresponding NAD^+^ analogues have been performed.^36,37^ However, evidence is currently lacking for the extent of intracellular conversion of precursor probes into the corresponding relevant metabolites, namely the intermediate adenosine monophosphate (AMP) and adenosine triphosphate (ATP) analogues, and the essential NAD^+^ analogue cofactor, the relative incorporation of these metabolite analogues into the corresponding native metabolite pools in cells and how their relative abundance in turn influences intracellular NAD^+^ analogue-mediated ADP-ribosylation.

Herein, we explore the suitability of a clickable adenosine-based ProTide proprobe, 6Yn-ProTide-Ad (6Yn-Pro), previously applied to study intracellular AMPylation^38^ as a potential precursor probe for intracellular labelling of ADP-ribosylated proteins. We look at the feasibility of this approach by measuring the metabolic flux of the precursor probe into key metabolite analogues in NAD^+^ biosynthesis, specifically 6Yn-ATP and 6Yn-NAD^+^, their relative incorporation into the native ATP and NAD^+^ pools in cells, respectively, and exploring conditions, from chemical supplementation to enzymatic overexpression, that favour intracellular NAD^+^ analogue-mediated ADP-ribosylation over other potential competing pathways, such as AMPylation. Through our efforts we demonstrate successful intracellular metabolic conversion of the precursor probe 6Yn-Pro into the ADP-ribosylation cofactor 6Yn-NAD^+^ by targeted metabolomics, and subsequent labelling and enrichment of auto-PARylated PARP1 and other known ADP-ribosylated proteins by proteomics. We provide the first evidence for the application of 6Yn-Pro for detection of intracellular protein ADP-ribosylation. In addition, we demonstrate, for the first time, that the NAD^+^ pathway can be targeted to boost intracellular NAD^+^ analogue biosynthesis through supplementation with an NAD^+^ precursor, reduced nicotinamide riboside (NRH), and through overexpression of a key enzyme in NAD^+^ biosynthesis, nicotinamide mononucleotide adenylyltransferase (NMNAT1); both are novel concepts that have not been previously explored in the context of clickable NAD^+^ analogue precursor probes for boosting intracellular NAD^+^ analogue biosynthesis. We also, for the first time, provide quantification of intracellular metabolic conversion of a clickable NAD^+^ analogue precursor probe, 6Yn-Pro, by measuring abundance levels of 6Yn-derived metabolites, 6Yn-AMP, 6Yn-ATP and 6Yn-NAD^+^. Furthermore, we successfully measure the relative % incorporation of 6Yn-ATP and 6Yn-NAD^+^ into the native cellular ATP and NAD^+^ pools, respectively, a concept that has not been previously explored for clickable NAD^+^ analogue precursor probes. We anticipate that the probe and described methodology will serve as a basis for further optimisation of the approach towards identification of novel intracellular ADP-ribosylated proteins, including non-canonical ADP-ribosylation events.^39^

## Results and discussion

**Fig. 1:**
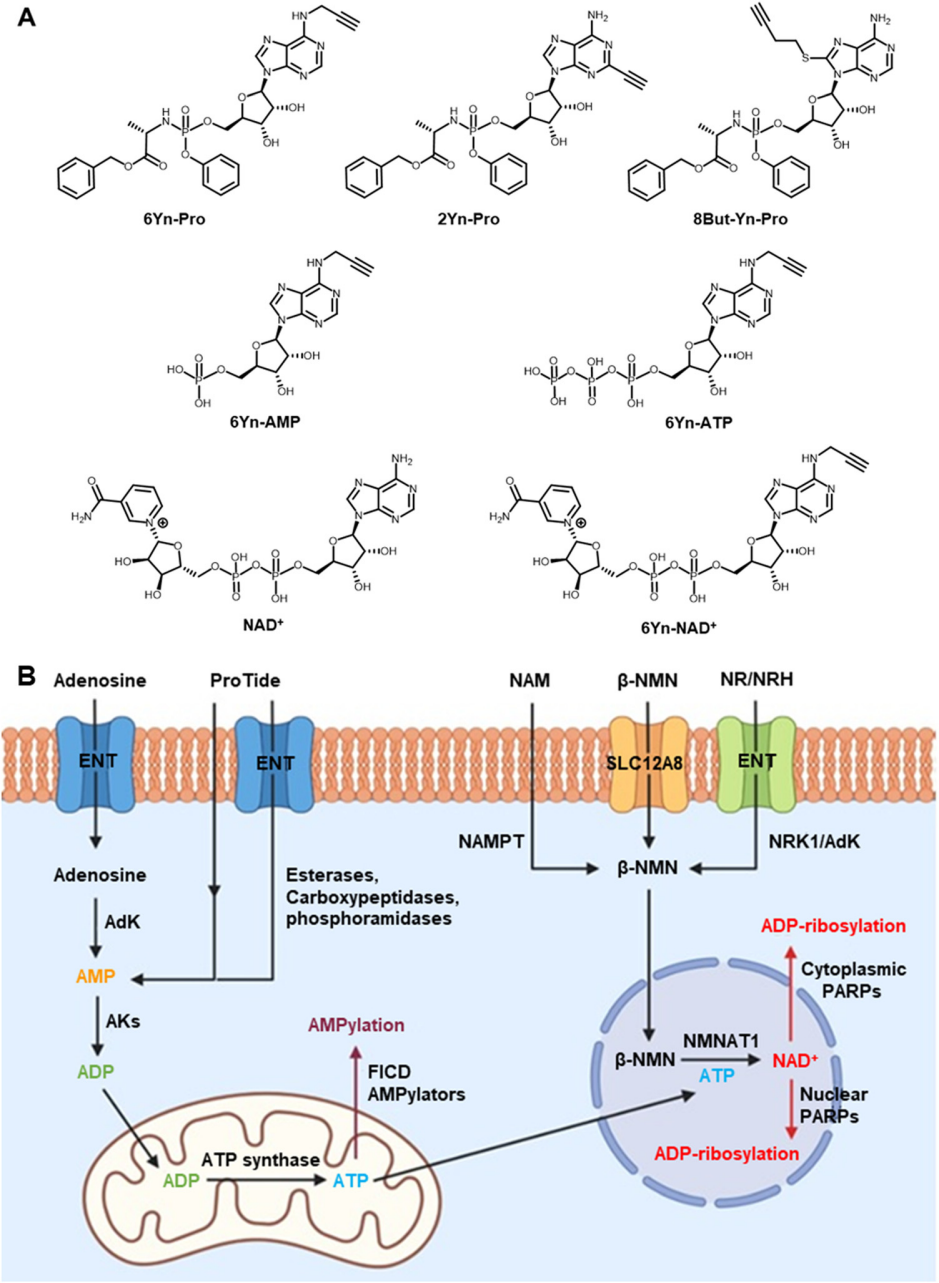
Chemical structures of probes and metabolites, and illustration of compartmentalisation of intracellular ATP, β-NMN and nuclear NAD^+^ biosynthesis. (A) Chemical structures of the three ProTide probes, 6Yn-Pro, 2Yn-Pro and 8But-Yn-Pro, and metabolites 6Yn-AMP, 6Yn-ATP, 6Yn-NAD^+^ and NAD^+^. (B) Extracellular adenosine (or ProTide-Ad proprobe) is transported into cells through equilibrative nucleoside transporters (ENTs) and converted into AMP by adenosine kinase (AdK), and then ADP by adenylate kinases (AKs), in the cytoplasm. ADP is subsequently transported into the mitochondria and converted into ATP by ATP synthase. ATP is then used in protein AMPylation by AMPylators, such as FICD, or transported into the nucleus for nuclear NAD^+^ biosynthesis *via* coupling to β-NMN by NMNAT1, and subsequent ADP-ribosylation by PARPs. β-NMN is biosynthesised from nicotinamide (NAM) by nicotinamide phosphoribosyltransferase (NAMPT), or through metabolic conversion of nicotinamide riboside (NR) or reduced nicotinamide riboside (NRH) by nicotinamide riboside kinase 1 (NRK1) or adenosine kinase (AdK), respectively. Extracellular β-NMN can also be transported intracellularly by cells expressing the β-NMN transporter channel SLC12A8.

6Yn-Pro (Fig. 1(A)) has previously been shown to undergo metabolic conversion into 6Yn-ATP in HeLa cells.^38^ In the final step of the NAD^+^ biosynthesis pathway (Fig. 1(B)), ATP and beta-nicotinamide mononucleotide (β-NMN) are coupled by nicotinamide mononucleotide adenylyltransferases (NMNAT1, 2, 3) to produce NAD^+^,^40^ and we hypothesised that 6Yn-ATP may also undergo intracellular metabolic conversion into the NAD^+^ analogue 6Yn-NAD^+^. Subsequently, PARPs might use metabolically generated 6Yn-NAD^+^ for intracellular protein ADP-ribosylation, in line with previous studies in cell lysates or with recombinant PARP1.^22–24^ We therefore sought to explore whether 6Yn-Pro could be used for successful intracellular labelling of ADP-ribosylated proteins. In addition, we sought to establish experimental conditions that promote metabolic conversion of 6Yn-ATP into 6Yn-NAD^+^ and thus favor protein ADP-ribosylation over competing AMPylation modification (Fig. S1A, ESI†) following probe treatment.

**Fig. 2:**
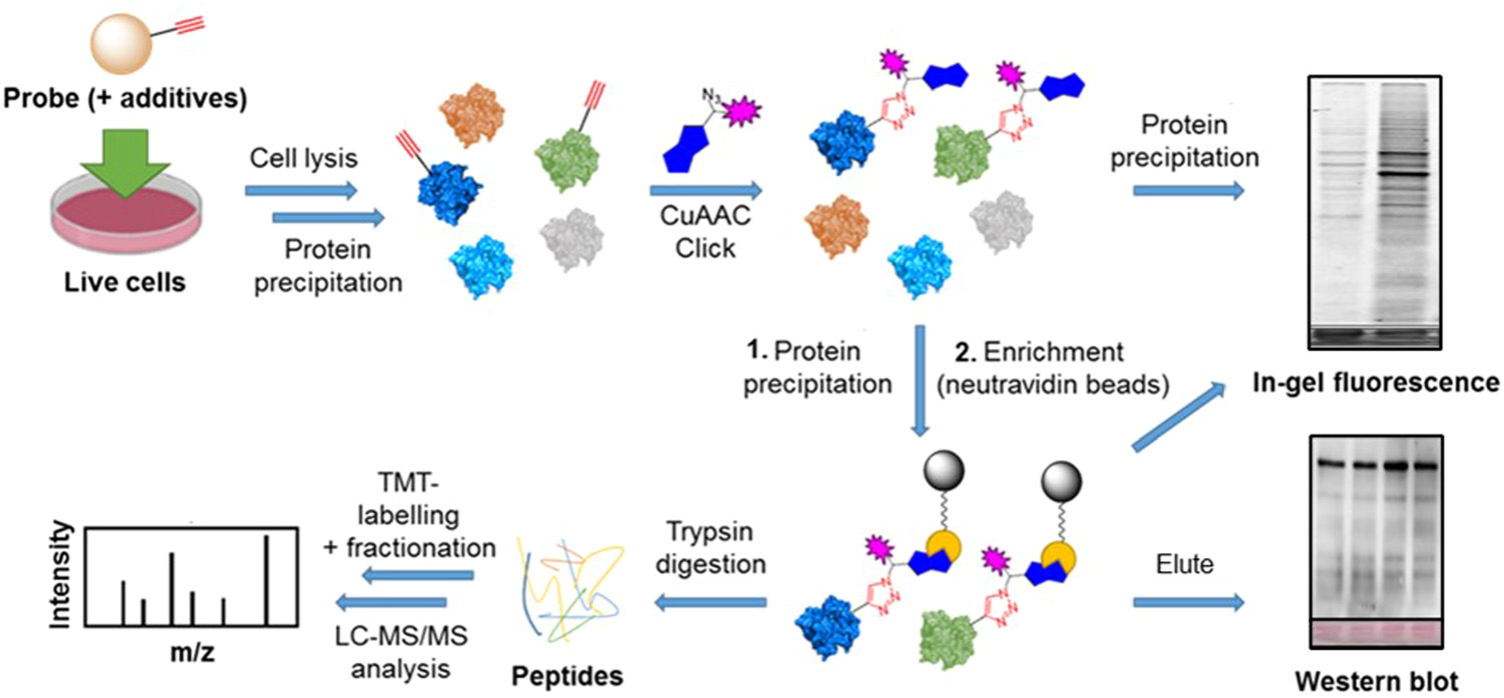
Overview of the workflow followed for sample preparation for analysis by in-gel fluorescence, western blot, and proteomics. Cell treatment with the relevant probe(s) and additive(s) is performed, typically for 24 h, followed by cell lysis and a protein precipitation step. Probe-labelled proteins are then captured by CuAAC click chemistry conjugation to a trifunctional azide-TAMRA-biotin capture reagent, allowing for visualisation of protein labelling *via* its fluorophore by IGF, or enrichment (pull-down) of labelled proteins *via* its biotin moiety by streptavidin affinity chromatography. For analysis by IGF and western blot, enriched proteins are eluted from the streptavidin beads by boiling; for proteomics analysis, on-bead digestion of the enrichment proteins is performed, followed by TMT-labelling for relative protein quantification, fractionation for increased protein coverage, and the corresponding peptides analysed by LC-MS/MS for protein identification.

In order to explore the structure–activity relationship among related adenosine probe analogues, 6Yn-Pro, along with two novel ProTide probes, 2Yn-ProTide-Ad (2Yn-Pro) and 8But-Yn-ProTide-Ad (8But-Yn-Pro), and the two adenosine analogues, 6Yn-Ad and 2Yn-Ad,^37^ were synthesised (Fig. 1(A); see Supplementary Methods, ESI†). Intracellular protein labelling was first investigated by in-gel fluorescence (IGF), by following the workflow outlined in Fig. 2. Following treatment of HEK293T and T47D cells with each probe or DMSO (negative control), the cells were lysed, proteins were precipitated using methanol:chloroform, and probe-labelled proteins were conjugated to azide-5-carboxytetramethylrhodamine (TAMRA)-biotin (AzTB; Fig. S1B, ESI†) by copper-catalysed azide–alkyne cycloaddition (CuAAC).

### Clickable adenosine-based probes non-specifically label proteins in lysates

Upon performing IGF experiments we discovered that the adenosine-based analogue probes 6Yn-Ad and 2Yn-Ad, including 6Yn-Pro, non-specifically interacted with proteins in denatured, enzymatically inactive lysates (of MDA-MB-231 cells), resulting in protein labelling being observed in the absence of metabolic and enzymatic activity (Fig. S2A, ESI†). The observed protein labelling pattern was very similar in probe-treated live MDA-MB-231 cells (Fig. S2B, ESI†), suggesting that non-specific protein labelling may have occurred in live-cell samples. These findings have important implications for downstream sample processing, including proteomics experiments where non-specifically labelled proteins may appear significantly enriched relative to untreated control samples, leading to false identification of protein targets. These findings also led us to hypothesise that upon cell treatment adenosine and ProTide-Ad probes enter cells and are released on cell lysis, possibly leading to non-enzyme mediated chemical conjugation. We discovered that adding a protein precipitation step in methanol:chloroform after cell lysis and prior to click chemistry conjugation decreased the levels of protein labelling (Fig. S2B and C, ESI†). Protein precipitation in methanol:chloroform is a highly efficient process with minimal loss of protein, as evidenced by Coomassie stained loading controls (Fig. S2B and C, ESI†), confirming that loss of protein labelling was not due to overall loss of protein loading following protein precipitation. The observed reduction could be explained by a potential reduction in non-specific protein labelling upon precipitation, possibly through removal of residual probe released from cells on lysis.

By following this approach, subsequent comparison of probe-treated and DMSO-treated cells after protein enrichment revealed extensive protein labelling in probe-treated samples (Fig. S3A and B, ESI†), confirming that the probes were metabolically converted into biologically active cofactors and subsequently attached to proteins, possibly as part of a PTM. Performing the precipitation step also demonstrated that 6Yn-Pro was superior in downstream incorporation into PTMs, and hence protein labelling, compared to adenosine analogues 6Yn-Ad and 2Yn-Ad. This was evidenced by the presence of protein bands in 6Yn-Pro-treated cells following precipitation (Fig. S2B, ESI†), in contrast to 6Yn-Ad and 2Yn-Ad-treated cells where protein labelling entirely disappeared following precipitation (Fig. S2C, ESI†), except for a single band at ∼75 kDa in 2Yn-Ad treated cells.

### Non-specific probe–protein interactions are facilitated by the ribose ring in adenosine

To uncover the origin of the non-specific probe–protein interactions, 6Yn-adenine was synthesised, and lysates and live cells were treated with this probe. 6Yn-adenine did not result in non-specific protein labelling in either case (Fig. S2B, ESI†), demonstrating that this phenomenon is facilitated by the ribose ring in adenosine. These findings potentially imply that nucleoside-based clickable probes other than adenosine might also suffer from this same drawback, which should be considered when using such probes in protein labelling experiments and warrants further investigation to confirm whether this phenomenon occurs with other clickable nucleoside probes. Although not further investigated in the present study, reversible cross-linking of the 2′ and 3′ hydroxyl groups of the ribose ring of the probes to carbonyl moieties in proteins could be a plausible mechanism for the reversible, non-enzymatic non-specific labelling of probe-treated samples.

### 6Yn-Pro is metabolically converted into ADP-ribosylation cofactor 6Yn-NAD^+^

**Fig. 3:**
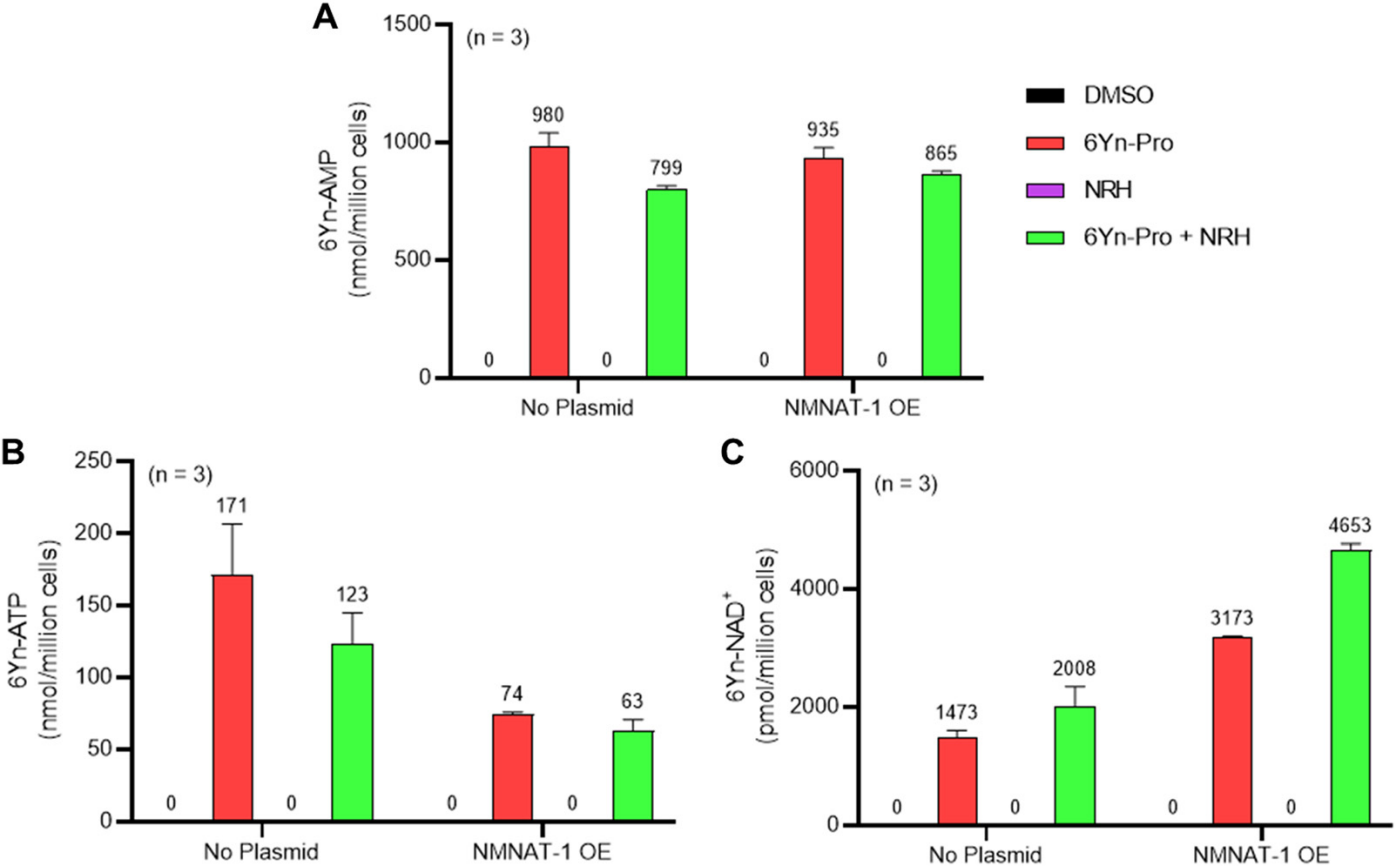
Metabolite profiling in HEK293T cells reveals intracellular conversion of 6Yn-Pro into 6Yn-ATP and 6Yn-NAD^+^. Metabolite profiling was performed following treatment with probe 6Yn-Pro (100 μM, 24 h), with/without NRH co-treatment (500 μM, 24 h), with/without NMNAT1 overexpression (24 h), or DMSO (negative control). Metabolite levels for (A) 6Yn-AMP, (B) 6Yn-ATP and (C) 6Yn-NAD^+^ were measured and quantified.

Since 6Yn-Pro has previously been shown to undergo metabolic conversion into 6Yn-ATP,^38^ we focused on exploring the extent of incorporation of 6Yn-ATP into the cellular ATP pool, whether it undergoes conversion into the analogue 6Yn-NAD^+^ and the resulting relative incorporation of 6Yn-NAD^+^ into the native NAD^+^ pool, by targeted metabolomics. To determine the extent of metabolic incorporation of 6Yn-Pro in NAD^+^ biosynthesis in cells, we employed targeted liquid chromatography-mass spectrometry (LC-MS) metabolomics^41^ (Fig. S4, ESI†) to measure the abundance of the key metabolites expected to arise following 24-hour treatment with 6Yn-Pro: 6Yn-AMP, 6Yn-ATP, and 6Yn-NAD^+^. We also explored the effect of supplementation with reduced nicotinamide riboside (NRH), a β-NMN/NMNH and NAD^+^ precursor, on 6Yn-NAD^+^ levels, which has previously been shown to increase intracellular NAD^+^ levels 3- to 10-fold in mammalian cell lines through metabolic conversion into β-NMNH.^42–44^ Finally, we investigated whether NMNAT1 overexpression (OE), responsible for conversion of ATP into NAD^+^ in the nucleus,^40^ resulted in increased 6Yn-NAD^+^ production. Analysis of extracted metabolites in cells treated only with 6Yn-Pro revealed the presence of all three metabolite analogues (Fig. 3(A)–(C)), demonstrating that the probe is cell-permeable, undergoes metabolic conversion into 6Yn-AMP (Fig. 3(A)), and is used to produce 6Yn-ATP (Fig. 3(B)) and 6Yn-NAD^+^ (Fig. 3(C)). Following 6Yn-Pro probe co-treatment with NRH, β-NMN levels increased ∼4-fold compared to 6Yn-Pro treatment alone (Fig. S5E, ESI†), whereas 6Yn-ATP levels decreased by 28%, (Fig. 3(B)) and 6Yn-NAD^+^ levels increased by 36%, (Fig. 3(C)). Collectively, the data indicate increased production of 6Yn-NAD^+^, possibly due to increased coupling of 6Yn-ATP and β-NMN/NMNH. We also measured the endogenous levels of ATP and NAD^+^ (Fig. S5A and B, ESI†) and calculated % incorporation of 6Yn-ATP and 6Yn-NAD^+^ in the endogenous ATP and NAD^+^ pools, which corresponded to 9.0% and 0.26% in the probe-only treated samples, respectively (Fig. S5C and D, ESI†). The observed disparity between % incorporation of 6Yn-ATP and 6Yn-NAD^+^ in their respective pools suggests that the final conversion step by NMNAT1 may be rate-limiting.

### NMNAT1 overexpression boosts intracellular production of 6Yn-NAD^+^

To explore whether higher NMNAT1 levels resulted in increased production of 6Yn-NAD^+^, NMNAT1 was overexpressed in HEK293T cells for 24 h prior to probe treatment (Fig. S6, ESI†). Although 6Yn-AMP levels were largely unaffected when NMNAT1 was overexpressed (Fig. 3(A)), 6Yn-ATP levels decreased by 57% and 49%, in NRH-untreated and NRH-co-treated cells, respectively, when compared to no NMNAT1 overexpression (Fig. 3(B)). Furthermore, 6Yn-NAD^+^ levels increased by 115% and 132%, in NRH-untreated and NRH-co-treated cells, respectively, when compared to cells without NMNAT1 overexpression (Fig. 3(C)). The drop in 6Yn-ATP levels and the increase in 6Yn-NAD^+^ levels following NMNAT1 overexpression imply increased production of 6Yn-NAD^+^ through increased coupling of the precursor 6Yn-ATP to β-NMN/NMNH, with the greatest effect observed when cells were also supplemented with NRH. Collectively, the data provide the first evidence that the 6Yn-ATP to 6Yn-NAD^+^ conversion step can be successfully targeted to increase metabolic production of 6Yn-NAD^+^ and raise the overall intracellular 6Yn-NAD^+^ levels.

### Metabolically produced 6Yn-NAD^+^ labels PARP1 and ADP-ribosylated proteins intracellularly

**Fig. 4:**
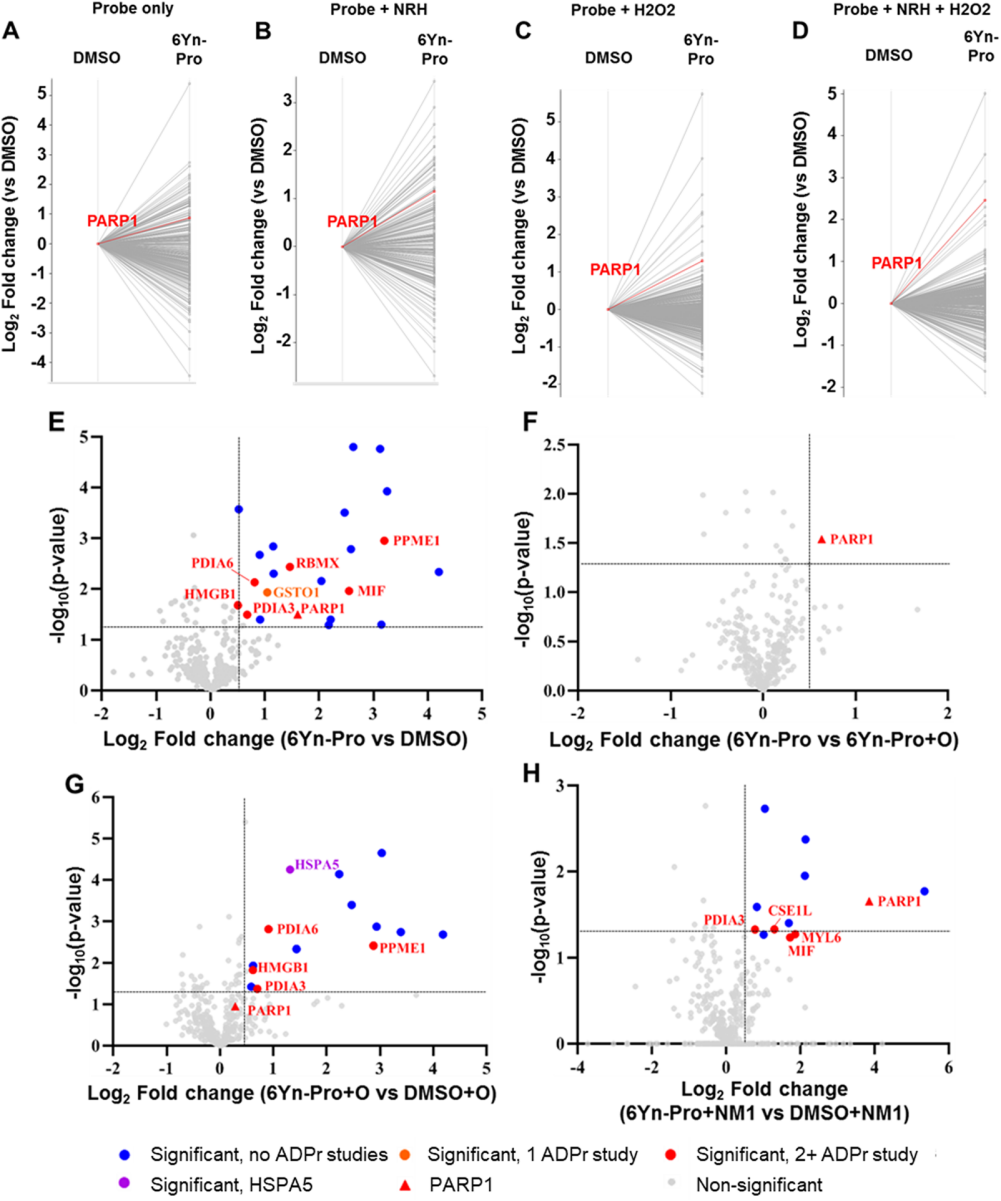
Proteomics analysis reveals protein labelling and enrichment of known ADPr proteins in 6Yn-Pro-treated HEK293T cells. Log_2_ Fold change represents the extent of protein enrichment/labelling in one treatment condition (typically probe treatment) relative to another. −log_10_(*p*-value) represents the statistical significance between replicates. (A)–(D) Profile plots showing protein labelling and enrichment, relative to the corresponding DMSO controls, following treatment of HEK293T cells with (A) 6Yn-Pro only (100 μM, 24 h), (B) 6Yn-Pro and NRH (500 μM, 24 h) co-treatment, (C) 6Yn-Pro followed by H_2_O_2_ treatment (in PBS, 2 mM, 6 min) and (D) 6Yn-Pro and NRH co-treatment followed by H_2_O_2_ treatment. Highlighted lines in red show PARP1 enrichment. (E)–(H) Volcano plots showing protein labelling and enrichment, and their statistical significance, for one treatment condition relative to another treatment condition. All samples were co-treated with NRH (500 μM, 24 h) followed by H_2_O_2_ treatment (in PBS, 2 mM, 6 min) prior to cell lysis. Significantly enriched known ADP-ribosylated proteins are shown in red, HSPA5 is shown in purple and all other significantly enriched proteins are shown in blue. Comparison of protein labelling in (E) 6Yn-Pro treated samples relative to DMSO control, (F) 6Yn-Pro treated samples relative to 6Yn-Pro and Olaparib co-treated samples, (G) 6Yn-Pro and Olaparib co-treated samples relative to DMSO and Olaparib co-treated samples and (H) 6Yn-Pro treated samples relative to DMSO control, following NMNAT1 overexpression (24 h).

Following confirmation that 6Yn-Pro undergoes intracellular metabolic conversion into 6Yn-NAD^+^, and IGF experiments confirming intracellular protein labelling, we next sought to identify labelled protein targets in 6Yn-Pro-treated HEK293T cells by proteomics. By following the workflow outlined in Fig. 2, comparison of protein enrichment was performed for probe-treated *vs.* DMSO-treated samples using TMT-labelling for relative protein quantification. We performed a preliminary experiment where HEK293T cells were treated with probe only, co-treated with probe and NRH, treated with probe and hydrogen peroxide (H_2_O_2_) just prior to cell lysis, or co-treated with probe, NRH and H_2_O_2_ (prior to cell lysis), and compared the protein labelling profiles to the corresponding DMSO controls (Fig. 4(A)–(D)). H_2_O_2_-treatment was included since ADP-ribosylation is known to be upregulated under oxidative stress/damage by PARP1/2-hyperactivation.^2^ We observed labelling and relative enrichment of PARP1 in all four conditions (Fig. 4(A)–(D)), demonstrating that metabolically generated 6Yn-NAD^+^, derived from 6Yn-Pro, is used by PARP1 for auto-PARylation. The extent of PARP1 labelling and enrichment was lowest in 6Yn-Pro only treated cells (absolute fold change = 1.8), and highest in cells co-treated with 6Yn-Pro, NRH and H_2_O_2_ (absolute Fold change = 5.5) (Table S1, ESI†). Similarly, the highest proportion of enriched proteins known to be ADP-ribosylated from previous studies was found in cells co-treated with 6Yn-Pro, NRH and H_2_O_2_ (51%), and lowest in 6Yn-Pro only treated cells (39%) (Table S2, ESI†). Mirroring the higher 6Yn-NAD^+^ levels observed in cells co-treated with 6Yn-Pro and NRH (Fig. 3(C) and Fig. S5D, ESI†), collectively, the data suggest that boosting 6Yn-NAD^+^ levels through supplementation with NRH promoted protein ADP-ribosylation with the analogue. In addition, these data support application of H_2_O_2_ prior to cell lysis to stimulate 6Yn-NAD^+^ incorporation by PARPs.

We next co-treated HEK293T cells with 6Yn-Pro and NRH and included an H_2_O_2_ treatment step prior to cell lysis in triplicate and quantified significantly enriched proteins in probe-treated *vs.* DMSO-treated samples (Fig. 4(E)).

A total of 22 proteins were significantly enriched (Table S3, ESI†), 8 of which are known ADP-ribosylated proteins, including PARP1 (Fig. 4(E)), by comparison to the ADP-ribosylation database generated by Buch-Larsen *et al.*^45^ (see ESI,† Table S3 in Buch-Larsen *et al.*;^45^ derived from the ADP-ribosylation (ADPr) data sets identified in the following 7 ADPr studies^45–51^) and Hendriks *et al.*^52^ (see Supplementary File Data 7, ESI† in Hendriks *et al.*^52^). A comparison of probe-treated *vs.* probe and Olaparib (O; PARP1/2 inhibitor) co-treated samples revealed that PARP1 was the only significantly enriched protein in the absence of Olaparib (Fig. 4(F), Table S4, ESI†). This suggested that the other 7 significantly enriched known ADP-ribosylated proteins (in orange/red, Fig. 4(E)) may be ADP-ribosylation targets of other PARPs, and the remaining 14 proteins (in blue) are potentially AMPylated proteins. In samples co-treated with Olaparib, we observed the appearance of HSPA5 (Table S5, ESI†), a known AMPylated protein,^53^ as a significantly enriched protein (Fig. 4(G)), which was not enriched in the absence of Olaparib. A possible explanation for this observation is that protein AMPylation (*via* 6Yn-ATP) is more likely when poly-ADP-ribosylation (and 6Yn-NAD^+^ consumption) is largely inhibited in the presence of Olaparib. These data suggest that AMPylation and ADP-ribosylation may compete for intermediates in the NAD^+^ synthesis pathway and illustrate the significance of optimising experimental conditions that favor production of the modified NAD^+^ cofactor and consequently protein ADP-ribosylation over AMPylation, when using an upstream precursor probe such as 6Yn-Pro.

### Protein ADP-ribosylation levels negatively correlate with high NMNAT1 expression levels

Based on the earlier observation that NMNAT1 overexpression resulted in increased levels of 6Yn-NAD^+^ (Fig. 3(C) and Fig. S5D, ESI†), we investigated the effect of NMNAT1 overexpression (NM1) on protein labelling and enrichment. HEK293T cells were transfected with NMNAT1 plasmid for 24 h prior to probe-treatment and then co-treated with 6Yn-Pro (or DMSO) and NRH for 24 h. All samples were treated with H_2_O_2_ prior to cell lysis. Comparison of probe-treated *vs.* DMSO-treated samples in the presence of NMNAT1 overexpression revealed that, compared to no NMNAT1 overexpression, only 12 (*vs.* 22) proteins were significantly enriched, and only 5 (*vs.* 8) of those were known ADP-ribosylated proteins (Table S6, ESI†), including PARP1 (Fig. 4(H)). Surprisingly, although the number of significantly enriched known ADP-ribosylated proteins decreased, the extent of PARP1 labelling and enrichment increased following NMNAT1 overexpression (log_2_ fold change = 4 *vs.* 1.6 without NMNAT1 overexpression), suggesting increased PARP1 auto-ADP-ribosylation. The former observation was contrary to our expectation that higher 6Yn-NAD^+^ levels observed following NMNAT1 overexpression Fig. 3(C) and Fig. S5D, ESI† would result in an increase in the number of significantly enriched known ADP-ribosylated proteins. However, the observation correlated well with overall MAR/PAR levels obtained by western blotting, which showed that the overall ADP-ribosylation levels decreased following NMNAT1 overexpression (Fig. S7, ESI†). These findings point to the presence of a negative feedback mechanism between high NMNAT1 expression and overall ADP-ribosylation. Optimisation of NMNAT1 levels warrants future investigation to explore whether intermediate levels of NMNAT1 lead to increased PARP1 activation without negatively impacting overall ADP-ribosylation levels.

### 6Yn-Pro probe enables identification of ADP-ribosylated proteins under oxidative stress

**Fig. 5:**
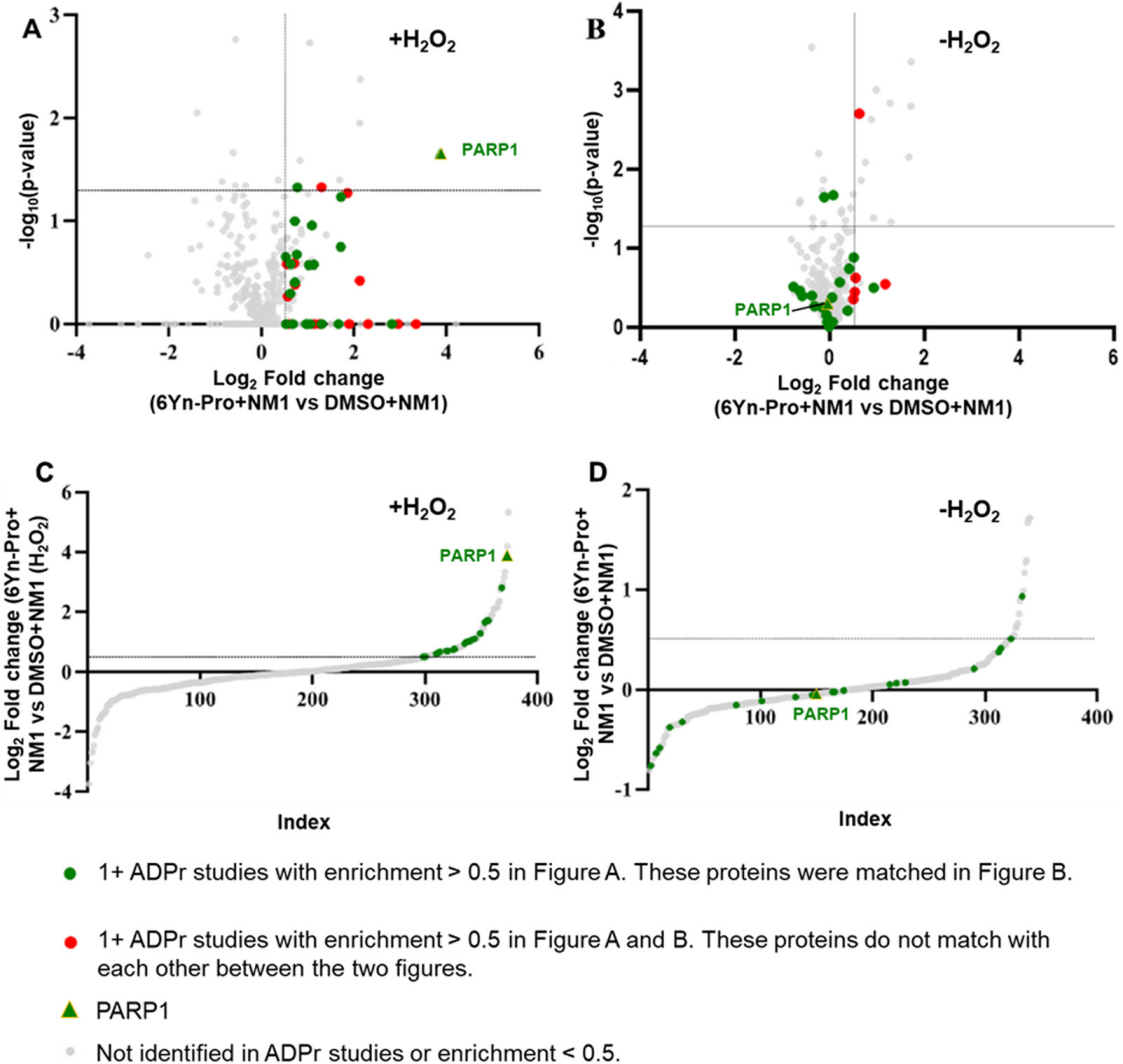
The presence of H_2_O_2_ upregulates labelling of ADPr proteins following treatment with 6Yn-Pro. Comparison of protein labelling profiles of 6Yn-Pro-treated HEK293T cells following NMNAT1 overexpression, (A) with H_2_O_2_ treatment and (B) without H_2_O_2_ treatment prior to cell lysis. All known ADP-ribosylated proteins with an enrichment value (log_2_ fold change) > 0.5 in A that matched the data set in B are highlighted in green. All known ADP-ribosylated proteins with an enrichment value (log_2_ fold change) > 0.5 in A that do not match the data set in B, and *vice versa*, are highlighted in red. (C) and (D) Comparison of the extent of enrichment of known ADP-ribosylated proteins in H_2_O_2_ treated samples and non-treated samples. (C) Data in volcano plot A visualised on an S-plot (significance is omitted). (D) Data in volcano plot B visualised on an S-plot (significance is omitted).

Since H_2_O_2_ significantly upregulates ADP-ribosylation, we determined the impact of H_2_O_2_ treatment on the enrichment of ADP-ribosylated proteins in probe-treated samples by following the workflow outlined in Fig. 2 and using TMT-labelling for relative protein quantification. In probe- and H_2_O_2_-treated samples (Fig. 4(H)), 35 known ADP-ribosylated proteins were significantly enriched (log_2_ fold change > 0.5; Fig. 5(A), (C) and Tables S7, S8, ESI†), 21 of which were also identified in the corresponding probe-treated samples in the absence of H_2_O_2_ treatment (Fig. 5(B), (D) and Tables S9, S10, ESI†). In the absence of H_2_O_2_, the enrichment of all 21 matching ADP-ribosylated proteins (highlighted in green) decreased, consistent with reduced ADP-ribosylation. By comparing the extent of enrichment of matching proteins in H_2_O_2_-treated and untreated samples, the same methodology could therefore be applied for the identification of novel ADP-ribosylation protein targets using the probe 6Yn-Pro.

## Conclusion

To our knowledge, the present study is the most comprehensive to date on the metabolic incorporation of cell-permeable precursor probes for the identification of intracellular ADP-ribosylation protein targets. We explore the feasibility of this approach by measuring the metabolic flux of the precursor probe 6Yn-Pro into NAD^+^ biosynthesis, and outline the importance of boosting relative NAD^+^ analogue levels relative to other probe metabolites that may be incorporated into competing PTMs, such as AMPylation. We demonstrate successful metabolic incorporation of clickable precursor probe 6Yn-Pro in the NAD^+^ biosynthesis pathway for intracellular metabolic production of the ADP-ribosylation cofactor 6Yn-NAD^+^, describe several experimental conditions for boosting intracellular 6Yn-NAD^+^ levels, and show its subsequent use for labelling and enrichment of intracellular ADP-ribosylated proteins. However, our western blot data (Fig. S3A and B, ESI†) suggest that validation of labelled proteins by antibody-specific methods may prove challenging or unfeasible due to the limitations of antibody recognition of analogue-modified proteins. We also identify an issue of non-specific labelling with nucleoside analogues and provide conditions to potentially reduce its impact. Through our comprehensive metabolomics analyses, we show the first evidence that the NAD^+^ biosynthesis pathway can be successfully targeted for boosting intracellular NAD^+^ analogue biosynthesis from a clickable NAD^+^ precursor probe (6Yn-Pro) by supplementation with the NAD^+^ precursor NRH and overexpression of NMNAT1, through specifically targeting the ATP to NAD^+^ conversion step, concepts that have not been previously explored. In addition, we successfully measure, for the first time, the abundance levels of 6Yn-ATP and 6Yn-NAD^+^ in cells, following 6Yn-Pro probe treatment, as well as native ATP and NAD^+^ levels, and thus calculate metabolite analogue % incorporation into native metabolite pools under different conditions. Our study therefore provides useful insights into the metabolic conversion of a clickable NAD^+^ analogue precursor probe in the different steps of the NAD^+^ biosynthesis pathway. It should be noted, however, that the relatively low conversion of 6Yn-Pro into 6Yn-NAD^+^ and its incorporation into the overall NAD^+^ pool may currently limit the extent of detecting intracellular ADP-ribosylation with the probe. Thus, we anticipate that these probes and methodology will serve as a basis for further optimisation of clickable NAD^+^ precursor probes for studying intracellular ADP-ribosylation events, including non-canonical ADP-ribosylation.^39^

## Methods

### Sample preparation for analysis by in-gel fluorescence and western blot

For IGF and WB experiments 500 000 cells were seeded per well in a 6-well plate in 2 mL of culture media for 24 h (37 °C, 5% CO_2_). Media was replaced with 1 mL of fresh culture media, and 1 μL of the appropriate 100 mM ProTide stock, 500 mM 6Yn-Ad or 500 mM 2Yn-Ad stock (in DMSO), or DMSO only, was then added to the respective wells (to 100 μM or 500 μM, respectively). Treatment was performed for 1 h or 24 h, unless otherwise stated. After incubation, cells were washed twice with PBS and lysed either with RIPA lysis buffer (25 mM Tris, 150 mM NaCl, 1% (v/v) Triton X100, 0.1% (w/v) SDS, 1% (w/v) sodium deoxycholate, protease inhibitor cocktail (1×, complete), benzonase (50 units per mL of buffer, 10 mM olaparib, pH 7.6)) or 4% (w/v) SDS lysis buffer (50 mM HEPES, 4% (w/v) SDS, 150 mM NaCl, protease inhibitor (1×), benzonase (50 units per mL of buffer), 10 mM olaparib) on ice. For generating denatured MDA-MB-231 lysates, MDA-MB-231 cells were lysed with the 4% (w/v) SDS lysis buffer for 1 h at r.t. Lysates were collected and cleared by centrifugation (16 000*g*, 10 min). For samples where protein precipitation was performed after cell lysis, and prior to click chemistry conjugation, protein precipitation was performed with MeOH : CHCl_3_ : H_2_O (4 : 1 : 2, v/v/v, where v = volume of sample). Proteins were pelleted by centrifugation (6000*g*, 5 min) and supernatants discarded. Pellets were washed with MeOH (500 μL × 3, 16 000*g*, 5 min), supernatants discarded, air-dried, and dissolved in 0.2% (w/v) SDS in PBS. Protein concentrations were measured using Bio-Rad DC Protein Assay kit. For samples where click chemistry conjugation by CuAAC was performed, a freshly prepared “click mix” was assembled by mixing the following stock reagents in a ratio 2 : 2 : 1 : 1 (v/v/v/v) – tris(2-carboxyethyl)phosphine hydroxide (TCEP, 50 mM in H_2_O), copper sulphate (CuSO_4_, 50 mM in H_2_O), tris(benzyltriazolylmethyl)amine (TBTA, 10 mM in DMSO) and the appropriate capture reagent (AzT or AzTB (both prepared in-house), 10 mM in DMSO). In a typical click reaction, 6 μL of the “click mix” was added to every 100 μL of protein sample (at a 1–2 mg mL^−1^ protein concentration), and a minimum of 100 μg of protein sample were labelled per reaction. Samples were briefly vortexed and the click reaction was allowed to proceed for 3 hours at r.t. with mild shaking. Subsequent quenching was performed by addition of 0.5 M EDTA_(aq)_ (final concentration of 5 mM), with shaking at r.t. for 2 min. Protein precipitation was subsequently performed, as previously described. Protein pellets were then dissolved in 0.2% (w/v) SDS in PBS to a final protein concentration of 1 mg mL^−1^, and 20 μg of protein per sample were used for analysis by IGF and WB.

### Sample preparation for analysis by proteomics

All proteomics experiments were performed with HEK293T cells in biological triplicates, unless otherwise stated. Cell seeding was performed for 24 hours in 6 cm dishes with 1 × 10^6^ cells seeded in 5 mL DMEM media (+10% (v/v) FBS) per dish (37 °C, 5% CO_2_). For cells where NMNAT1 overexpression was induced, transfection with the CMV NMNAT1 plasmid (1000 ng per mL of media) was performed for 24 h, prior to probe treatment (see ESI†). Prior to metabolic labelling, culture media was replaced with 3 mL of fresh media. 3 μL of the appropriate 100 mM ProTide stock (in DMSO), or DMSO only, was then added to the respective dish (to 100 μM) and treatment was performed for 24 h. For cells co-treated with NRH, 3 μL of a 500 mM NRH stock (in DMSO) was added to the appropriate dish at the same time as the ProTide and incubated for 24 h. Subsequently, cells were washed with PBS in the following manner: for cells (to be) treated with 2 mM H_2_O_2_ (in PBS), cells were washed once with PBS (5 mL) and then incubated with 2 mM H_2_O_2_ (in PBS) at 37 °C for 6 min prior to lysis. For cells not treated with H_2_O_2_, cells were washed twice with PBS (5 mL) and directly lysed. Lysates were generated with RIPA lysis buffer (described above) and incubation on ice for 15 min. Clearing of the lysates was performed by centrifugation at 13 000*g* at 4 °C for 5 min and protein precipitation of the lysates was performed with MeOH:H_2_O:CHCl_3_, as previously described. Protein pellets were dissolved in 0.2% (w/v) SDS in PBS (500 μL per sample) and protein concentrations were measured using the Bio-Rad DC Protein Assay kit. CuAAC click reaction was performed using 500–1000 μg of protein per sample (2 mg mL^−1^ final concentration) and AzB (Sigma-Aldrich, 762074) or AzTB (prepared in-house) for 2 hours at r.t., as described in the previous section. Following incubation and quenching of the reaction, protein precipitation was performed and pelleted proteins were dissolved in 0.2% SDS in PBS (500 μL or 1000 μL per sample, respectively, to a 1 mg mL^−1^ final concentration). Protein pull-down was then performed by incubating the 500–1000 μg (500–1000 μL, 1 mg mL^−1^) of clicked protein sample (0.2% (w/v) SDS in PBS pH 7.4) with 50–100 μL Neutravidin agarose beads (Thermo Scientific Pierce™) for 3 hours at r.t., with shaking (750–800 rpm). Prior to the pull-down incubation, the Neutravidin agarose beads were washed five times with 0.2% (w/v) SDS in PBS, pelleted by centrifugation (3500*g*, 2 min) and supernatant aspirated by pipetting. Beads were resuspended to their original volume and the appropriate volume of beads added to each sample (1 : 10 beads volume : sample volume). Following the incubation, the bead samples were pelleted by centrifugation (3500*g*, 3 min), supernatants aspirated, samples washed with 0.2% (w/v) SDS in 50 mM HEPES (pH 8.0) four times (1 mL each) and then washed three more times with 50 mM HEPES (pH 8.0). Each sample was subsequently made up to a volume of ∼200 μL by addition of 150 μL 50 mM HEPES (pH 8.0) and on-bead sample reduction and alkylation was performed in the following manner: stock solutions of TCEP (50 mM) and 2-chloroacetamide (CAA, 150 mM) were prepared in 50 mM HEPES (pH 8.0). Equal volumes of the stocks were combined and 40 μL of this mixture were added to each sample, giving final concentrations of 5 mM (TCEP) and 15 mM (CAA). Samples were then incubated at r.t. with shaking for 15 min and peptides from each sample were released from the beads by digestion with trypsin. For trypsin digestion, 5 μL of trypsin stock (0.2 μg μL^−1^) were added to each sample and subsequently incubated for 16–18 hours at 37 °C with shaking (900–1000 rpm). Digestion was quenched by the addition of EDTA-free protease inhibitor cocktail (4 μL, 50×) and incubation for 5 min at r.t. with shaking. Beads were pelleted by centrifugation (3500*g*, 3 min) and supernatants were transferred into clean Eppendorf tubes. Each beads sample was washed once with 50 mM HEPES (pH 8.0, 100 μL), beads were pelleted (3500*g*, 3 min) and each washing combined with its respective supernatant from the first centrifugation step. Peptide concentration was measured for each sample using the Pierce^TM^ Quantitative Fluorometric Peptide Assay (Thermo Scientific), in accordance with the manufacturer's protocol, and TMT-labelling was performed subsequently followed by fractionation (see Supplementary Biological Methods, ESI†).

## Conflicts of interest

The authors declare no conflict of interest.

## Supplementary Material

CB-005-D4CB00043A-s001

CB-005-D4CB00043A-s002

CB-005-D4CB00043A-s003

## References

[cit1] Gibson B. A., Kraus W. L. (2012). New insights into the molecular and cellular functions of poly(ADP-ribose) and PARPs. Nat. Rev. Mol. Cell Biol..

[cit2] Hoch N. C., Polo L. M. (2020). ADP-ribosylation: from molecular mechanisms to human disease. Genet. Mol. Biol..

[cit3] Vyas S., Matic I., Uchima L., Rood J., Zaja R., Hay R. T., Ahel I., Chang P. (2014). Family-wide analysis of poly(ADP-ribose) polymerase activity. Nat. Commun..

[cit4] Vivelo C. A., Leung A. K. (2015). Proteomics approaches to identify mono-(ADP-ribosyl)ated and poly(ADP-ribosyl)ated proteins. Proteomics.

[cit5] Liu C., Yu X. (2015). ADP-Ribosyltransferases and Poly ADP-Ribosylation. Curr. Protein Pept. Sci..

[cit6] Langelier M. F., Planck J. L., Roy S., Pascal J. M. (2012). Structural basis for DNA damage-dependent poly(ADP-ribosyl)ation by human PARP-1. Science.

[cit7] Liu C., Vyas A., Kassab M. A., Singh A. K., Yu X. (2017). The role of poly ADP-ribosylation in the first wave of DNA damage response. Nucleic Acids Res..

[cit8] Pascal J. M. (2018). The comings and goings of PARP-1 in response to DNA damage. DNA Repair.

[cit9] Bock F. J., Todorova T. T., Chang P. (2015). RNA Regulation by Poly(ADP-Ribose) Polymerases. Mol. Cell.

[cit10] Daugherty M. D., Young J. M., Kerns J. A., Malik H. S. (2014). Rapid evolution of PARP genes suggests a broad role for ADP-ribosylation in host-virus conflicts. PLoS Genet..

[cit11] Xing J., Zhang A., Du Y., Fang M., Minze L. J., Liu Y. J., Li X. C., Zhang Z. (2021). Identification of poly(ADP-ribose) polymerase 9 (PARP9) as a noncanonical sensor for RNA virus in dendritic cells. Nat. Commun..

[cit12] Guo T., Zuo Y., Qian L., Liu J., Yuan Y., Xu K., Miao Y., Feng Q., Chen X., Jin L., Zhang L., Dong C., Xiong S., Zheng H. (2019). ADP-ribosyltransferase PARP11 modulates the interferon antiviral response by mono-ADP-ribosylating the ubiquitin E3 ligase β-TrCP. Nat. Microbiol..

[cit13] Li L., Zhao H., Liu P., Li C., Quanquin N., Ji X., Sun N., Du P., Qin C. F., Lu N., Cheng G. (2018). PARP12 suppresses Zika virus infection through PARP-dependent degradation of NS1 and NS3 viral proteins. Sci. Signaling.

[cit14] Grunewald M. E., Chen Y., Kuny C., Maejima T., Lease R., Ferraris D., Aikawa M., Sullivan C. S., Perlman S., Fehr A. R. (2019). The coronavirus macrodomain is required to prevent PARP-mediated inhibition of virus replication and enhancement of IFN expression. PLoS Pathog..

[cit15] Vermehren-Schmaedick A., Huang J. Y., Levinson M., Pomaville M. B., Reed S., Bellus G. A., Gilbert F., Keren B., Heron D., Haye D., Janello C., Makowski C., Danhauser K., Fedorov L. M., Haack T. B., Wright K. M., Cohen M. S. (2021). Characterization of PARP6 Function in Knockout Mice and Patients with Developmental Delay. Cells.

[cit16] Ossovskaya V., Koo I. C., Kaldjian E. P., Alvares C., Sherman B. M. (2010). Upregulation of Poly (ADP-Ribose) Polymerase-1 (PARP1) in Triple-Negative Breast Cancer and Other Primary Human Tumor Types. Genes Cancer.

[cit17] Wang L., Liang C., Li F., Guan D., Wu X., Fu X., Lu A., Zhang G. (2017). PARP1 in Carcinomas and PARP1 Inhibitors as Antineoplastic Drugs. Int. J. Mol. Sci..

[cit18] Yang H. Y., Shen J. X., Wang Y., Liu Y., Shen D. Y., Quan S. (2019). Tankyrase Promotes Aerobic Glycolysis and Proliferation of Ovarian Cancer through Activation of Wnt/β-Catenin Signaling. BioMed Res. Int..

[cit19] Yao N., Chen Q., Shi W., Tang L., Fu Y. (2019). PARP14 promotes the proliferation and gemcitabine chemoresistance of pancreatic cancer cells through activation of NF-κB pathway. Mol. Carcinog..

[cit20] Schleicher E. M., Galvan A. M., Imamura-Kawasawa Y., Moldovan G. L., Nicolae C. M. (2018). PARP10 promotes cellular proliferation and tumorigenesis by alleviating replication stress. Nucleic Acids Res..

[cit21] Zhang J., Snyder S. H. (1993). Purification of a nitric oxide-stimulated ADP-ribosylated protein using biotinylated beta-nicotinamide adenine dinucleotide. Biochemistry.

[cit22] Du J., Jiang H., Lin H. (2009). Investigating the ADP-ribosyltransferase activity of sirtuins with NAD analogues and 32P-NAD. Biochemistry.

[cit23] Jiang H., Kim J. H., Frizzell K. M., Kraus W. L., Lin H. (2010). Clickable NAD analogues for labeling substrate proteins of poly(ADP-ribose) polymerases. J. Am. Chem. Soc..

[cit24] Buntz A., Wallrodt S., Gwosch E., Schmalz M., Beneke S., Ferrando-May E., Marx A., Zumbusch A. (2016). Real-Time Cellular Imaging of Protein Poly(ADP-ribos)ylation. Angew. Chem., Int. Ed..

[cit25] Wallrodt S., Simpson E. L., Marx A. (2017). Investigation of the action of poly(ADP-ribose)-synthesising enzymes on NAD^+^ analogues. Beilstein J. Org. Chem..

[cit26] Lehner M., Rieth S., Höllmüller E., Spliesgar D., Mertes B., Stengel F., Marx A. (2022). Profiling of the ADP-Ribosylome in Living Cells. Angew. Chem., Int. Ed..

[cit27] Wallrodt S., Buntz A., Wang Y., Zumbusch A., Marx A. (2016). Bioorthogonally Functionalized NAD(+) Analogues for In-Cell Visualization of Poly(ADP-Ribose) Formation. Angew. Chem., Int. Ed..

[cit28] Wang Y., Rösner D., Grzywa M., Marx A. (2014). Chain-terminating and clickable NAD^+^ analogues for labeling the target proteins of ADP-ribosyltransferases. Angew. Chem., Int. Ed..

[cit29] Zhang X. N., Cheng Q., Chen J., Lam A. T., Lu Y., Dai Z., Pei H., Evdokimov N. M., Louie S. G., Zhang Y. (2019). A ribose-functionalized NAD^+^ with unexpected high activity and selectivity for protein poly-ADP-ribosylation. Nat. Commun..

[cit30] Carter-O'Connell I., Jin H., Morgan R. K., David L. L., Cohen M. S. (2014). Engineering the substrate specificity of ADP-ribosyltransferases for identifying direct protein targets. J. Am. Chem. Soc..

[cit31] Carter-O'Connell I., Jin H., Morgan R. K., Zaja R., David L. L., Ahel I., Cohen M. S. (2016). Identifying Family-Member-Specific Targets of Mono-ARTDs by Using a Chemical Genetics Approach. Cell Rep..

[cit32] Carter-O'Connell I., Vermehren-Schmaedick A., Jin H., Morgan R. K., David L. L., Cohen M. S. (2018). Combining Chemical Genetics with Proximity-Dependent Labeling Reveals Cellular Targets of Poly(ADP-ribose) Polymerase 14 (PARP14). ACS Chem. Biol..

[cit33] Rodriguez K. M., Buch-Larsen S. C., Kirby I. T., Siordia I. R., Hutin D., Rasmussen M., Grant D. M., David L. L., Matthews J., Nielsen M. L., Cohen M. S. (2021). Chemical genetics and proteome-wide site mapping reveal cysteine MARylation by PARP-7 on immune-relevant protein targets. eLife.

[cit34] Gibson B. A., Zhang Y., Jiang H., Hussey K. M., Shrimp J. H., Lin H., Schwede F., Yu Y., Kraus W. L. (2016). Chemical genetic discovery of PARP targets reveals a role for PARP-1 in transcription elongation. Science.

[cit35] Palavalli Parsons L. H., Challa S., Gibson B. A., Nandu T., Stokes M. S., Huang D., Lea J. S., Kraus W. L. (2021). Identification of PARP-7 substrates reveals a role for MARylation in microtubule control in ovarian cancer cells. eLife.

[cit36] Westcott N. P., Fernandez J. P., Molina H., Hang H. C. (2017). Chemical proteomics reveals ADP-ribosylation of small GTPases during oxidative stress. Nat. Chem. Biol..

[cit37] Kalesh K., Lukauskas S., Borg A. J., Snijders A. P., Ayyappan V., Leung A. K. L., Haskard D. O., DiMaggio P. A. (2019). An Integrated Chemical Proteomics Approach for Quantitative Profiling of Intracellular ADP-Ribosylation. Sci. Rep..

[cit38] Kielkowski P., Buchsbaum I. Y., Kirsch V. C., Bach N. C., Drukker M., Cappello S., Sieber S. A. (2020). FICD activity and AMPylation remodelling modulate human neurogenesis. Nat. Commun..

[cit39] Ahel I., Schuller M. (2022). Beyond protein modification: the rise of non-canonical ADP-ribosylation. Biochem. J..

[cit40] Ryu K. W., Nandu T., Kim J., Challa S., DeBerardinis R. J., Kraus W. L. (2018). Metabolic regulation of transcription through compartmentalized NAD^+^ biosynthesis. Science.

[cit41] Roberts L. D., Souza A. L., Gerszten R. E., Clish C. B. (2012). Targeted metabolomics. Curr. Protoc. Mol. Biol..

[cit42] Yang Y., Mohammed F. S., Zhang N., Sauve A. A. (2019). Dihydronicotinamide riboside is a potent NAD^+^ concentration enhancer *in vitro* and *in vivo*. J. Biol. Chem..

[cit43] Giroud-Gerbetant J., Joffraud M., Giner M. P., Cercillieux A., Bartova S., Makarov M. V., Zapata-Pérez R., Sánchez-García J. L., Houtkooper R. H., Migaud M. E., Moco S., Canto C. (2019). A reduced form of nicotinamide riboside defines a new path for NAD^+^ biosynthesis and acts as an orally bioavailable NAD^+^ precursor. Mol. Metab..

[cit44] Yang Y., Zhang N., Zhang G., Sauve A. A. (2020). NRH salvage and conversion to NAD^+^ requires NRH kinase activity by adenosine kinase. Nat. Metab..

[cit45] Buch-Larsen S. C., Hendriks I. A., Lodge J. M., Rykær M., Furtwängler B., Shishkova E., Westphall M. S., Coon J. J., Nielsen M. L. (2020). Mapping Physiological ADP-Ribosylation Using Activated Ion Electron Transfer Dissociation. Cell Rep..

[cit46] Zhang Y., Wang J., Ding M., Yu Y. (2013). Site-specific characterization of the Asp- and Glu-ADP-ribosylated proteome. Nat. Methods.

[cit47] Larsen S. C., Hendriks I. A., Lyon D., Jensen L. J., Nielsen M. L. (2018). Systems-wide Analysis of Serine ADP-Ribosylation Reveals Widespread Occurrence and Site-Specific Overlap with Phosphorylation. Cell Rep..

[cit48] Hendriks I. A., Larsen S. C., Nielsen M. L. (2019). An Advanced Strategy for Comprehensive Profiling of ADP-ribosylation Sites Using Mass Spectrometry-based Proteomics. Mol. Cell. Proteomics.

[cit49] Bilan V., Leutert M., Nanni P., Panse C., Hottiger M. O. (2017). Combining Higher-Energy Collision Dissociation and Electron-Transfer/Higher-Energy Collision Dissociation Fragmentation in a Product-Dependent Manner Confidently Assigns Proteomewide ADP-Ribose Acceptor Sites. Anal. Chem..

[cit50] Jungmichel S., Rosenthal F., Altmeyer M., Lukas J., Hottiger M. O., Nielsen M. L. (2013). Proteome-wide identification of poly(ADP-Ribosyl)ation targets in different genotoxic stress responses. Mol. Cell.

[cit51] Martello R., Leutert M., Jungmichel S., Bilan V., Larsen S. C., Young C., Hottiger M. O., Nielsen M. L. (2016). Proteome-wide identification of the endogenous ADP-ribosylome of mammalian cells and tissue. Nat. Commun..

[cit52] Hendriks I. A., Buch-Larsen S. C., Prokhorova E., Elsborg J. D., Rebak A. K. L. F. S., Zhu K., Ahel D., Lukas C., Ahel I., Nielsen M. L. (2021). The regulatory landscape of the human HPF1- and ARH3-dependent ADP-ribosylome. Nat. Commun..

[cit53] Ham H., Woolery A. R., Tracy C., Stenesen D., Kramer H., Orth K. (2014). Unfolded protein response-regulated Drosophilia Fic (dFic) protein reversibly AMPylates BiP chaperone during endoplasmic reticulum homeostasis. J. Biol. Chem..

